# Effect of platelet-rich plasma combined with tranexamic acid in the treatment of melasma and its effect on the serum levels of vascular endothelial growth factor, endothelin-1 and melatonin

**DOI:** 10.12669/pjms.38.8.6786

**Published:** 2022

**Authors:** Ci Zhang, Tianpeng Wu, Nan Shen

**Affiliations:** 1 Ci Zhang, Department of Cosmetic Dermatology, Jinan Airong Plastic Surgery Hospital, 319 Jingqi Road, Jinan, Shandong 250001, P.R. China; 2 Tianpeng Wu Department of Medical, Shenyang Aerospace Hospital, 12 Xindongyi Street, Shenyang, Liaoning 110043, P.R. China; 3 Nan Shen Department of Dermatology, Shanxi Maternal and Child Health Hospital, 13 Xinmin North Street, Taiyuan, Shanxi 030006, P.R. China

**Keywords:** Melasma, Platelet-rich plasma, Tranexamic acid, Serum level

## Abstract

**Objective:**

To investigate the effect of platelet-rich plasma (PRP) combined with tranexamic acid (TXA) in the treatment of melasma and its effect on the serum levels of vascular endothelial growth factor (VEGF), endothelin-1 (ET-1) and melanin stimulating hormone (MSH).

**Methods:**

We retrospectively analyzed clinical data of 80 patients with melasma treated in our hospital from January 2020 to June 2021. Patients (n=38) in the control group received simple oral TXA treatment. Patients (n=42) in the study group received PRP combined with oral TXA treatment. We assessed the treatment effects on the serum biochemical index levels, the adverse reactions, and the recurrence rates in the two groups.

**Results:**

The total efficacy of the study group (90.48%) was higher than that of the control group (73.68%) (*p*<0.05). After the treatment, the levels of serum VEGF increased and the levels of ET-1 and MSH decreased in both groups, but the changes in the study group were more pronounced than those in the control group (*p*<0.05). We found similar incidences of the adverse reaction in the study group (7.14%) and the control group (5.26%; *p*>0.05). The disease recurrence rates between the two groups three months after the treatment were similar (*p*>0.05). However, the disease recurrence rate in the study group (4.76%) was lower than that in the control group (21.05%) 6 months after treatment (*p*<0.05).

**Conclusions:**

PRP combined with oral TXA can improve the treatment effect of TXA alone in the treatment of melasma, maintaining normal levels of VEGF, ET-1 and MSH, reducing disease recurrences.

## INTRODUCTION

Melasma is a common dermatological condition characterized by abnormal melanin increases within the dermis and epidermis. The incidence rate of melasma is higher in middle-aged and young women.^1^ Melasma spots appear mostly on the upper lip, nose, forehead, cheeks, and other sun-exposed areas. The light tan or yellowish-brown spots affect the patients’ self-aesthetic perception negatively impacting their mental health.^2^ The pathogenesis of melasma is complex, and is closely related to sunlight exposure, pregnancy, and the application of cosmetics.^3^ Melasma’s patches and spots can be difficult to treat, requiring long treatment cycles and recurring easily. Therefore, research on effective and safe melasma treatments remains important.^4^

Study results have shown that tranexamic acid (TXA) is one of the more effective treatments against melasma.^5^,^6^ Platelet-rich plasma (PRP) is a platelet concentrate extracted from individual’s whole blood. It contains many growth factors that can help repair the skin. PRP is considered a promising treatment for acne and hyperpigmentation.^7^ However, few systematic studies have been conducted on the value of combined application of TXA and PRP against melasma. In this study, we explored the outcomes of a treatment for melasma combining TXA and PRP.

## METHODS

We extracted data from the records of 80 patients with melasma treated in our hospital from January 2020 to June 2021 (10 males and 70 females). A control group included data from 38 patients treated with oral TXA alone, and the study group contained data from 42 patients treated with PRP and oral TXA.

### Inclusion criteria:

- The melasma diagnosis met the diagnostic criteria^8^
- The patient’s face was light brown to dark brown, with clearly defined patches, mostly symmetrically distributed, without scales or inflammation
- The patient’s record had complete clinical data

### Exclusion criteria:

- Patients with dysfunctions of kidneys, liver, heart, or other organs
- Lactating or pregnant women
- Patients with neurological diseases
- History of smoking or drinking

### Ethical approval:

This study was approved by the ethics committee of our hospital (Approval number 21136, Date: 2021-10-15).

### Tranexamic acid treatment:

One 250-mg oral tablet in the morning and one in evening for three months (Shanghai Xinyi Vientiane Pharmaceutical, H31020040, 250 mg/tablet, 30 tablets/box).

***PRP treatment***: We drew 10 mL of the patient’s own venous blood and gently inverted the tube and shook it evenly before centrifuging at 3500 rpm for 10 minutes. We let the tube stand for five minutes, then inverted the preparation tube, and suspended the cell concentrate on the isolation layer to form the PRP before extracting it for the water light injection treatment. The specific process included cleaning the skin of the affected area, evenly applying lidocaine cream (about 3-5 mm thick) covering the area for about 60 minutes to keep a fresh film of anesthetic in contact with the skin, cleaning the cream, disinfecting and deionizing the skin, putting a sterile film coated water light gun head on the injection instrument, adjusting the instrument mode to very fast, giving a fixed dose of 0.0179 to 0.0208 mL, and injecting the PRP under the melasma lesion. PRP is the whole face injection treatment, once a month, a total of three times.^9^ Equipment used: Regen ACRC (Regen PRP, Regenlab, Switzerland); centrifuge (Micro 17; Thermo Fisher Scientific); water light injection instrument (batch number PD3-INJ 5440; model, PANACE-DS-30, Demasa instrument factory, Korea).

### Treatment effects:

Two professional dermatologists evaluated the skin lesions of each patient before and after the treatment course according to the international MASI (melasma area and severity index) evaluation standard using the following equations: A decreases in the MASI = (pre-treatment value - post-treatment value)/pre-treatment value×100%. We classified patients with decreases of the MASI higher than 90% as having a successful treatment; those with a MASI decrease higher than 60% but lower than 90% as having a moderate improvement; those with a MASI decrease higher than 20% but lower than 60% as showing mild improvement; and those with a MASI decrease lower than 20% as having an unsuccessful treatment. We calculated the total efficacy by adding the percentages of successful treatment, moderate improvement, and mild improvement effects.^10^

### Serum biochemical index levels:

We centrifuged 4-ml fasting samples of elbow vein blood at 3500 rpm for 10 minutes, then took the supernatants, and determined its serum contents of VEGF, ET-1 and MSH using an enzyme-linked immunosorbent assay kit purchased from DRG (Germany) and following the manufacturer’s instructions.

### Safety:

We evaluated and recorded the incidence of adverse reactions during the treatment of the two groups including instances of depigmentation, flushing and scarring.

### Recurrence rate:

We recorded the disease recurrence rates three and six months after treatment.

### Statistical analysis:

Spss22.0 software was used to process the data. The measurement data were represented by (X̄±*s*), the inter group comparisons were subjected to independent sample *t*-tests, and the intra group comparisons were subjected to paired *t*-tests. The count data were represented by [*n*(%)], and analyzed by *χ^2^* test. We considered all differences with *p* < 0.05 as statistically significant.

## RESULTS

In all, 80 patients met the inclusion criteria, 38 received oral TXA alone and 42 received PRP combined with TXA. The patients in both groups had similar values for gender, age, course of disease and disease location (*p*>0.05; Table-I). The total efficacy of the study group (90.48%) was higher than that of the control group (73.68%; *p* < 0.05; Table-II).

**Table-I:** Comparison of basic clinical data between the two groups [(X̄±*S* ), n(%)].

Group	*n*	Men/ Women	Age (years)	Course of disease (years)	Disease site		

					Center of cheek	Frontotemporal	Mandible
Study group	42	6/36	29.19±4.83	5.45±3.03	36 (85.71)	3 (7.14)	3 (7.14)
Control group	38	4/34	28.65±4.64	4.94±2.99	32 (84.21)	5 (13.16)	1 (2.63)
χ^2^/ t		0.258	0.501	0.747	1.539		
p		0.612	0.617	0.457	0.463		

**Table-II:** Comparison of treatment effects between the two groups [*n* (%)].

Group	*n*	Successful treatment	Moderate improvement effect	Mild improvement	Unsuccessful treatment	Total effective rate
Study group	42	16 (38.10)	17 (40.48)	5 (11.90)	4 (9.52)	38 (90.48)
Control group	38	9 (23.68)	12 (31.58)	7 (18.42)	10 (26.32)	28 (73.68)
χ^2^						3.896
p						0.048

We found similar levels of serum VEGF, ET-1 and MSH between the two groups before the treatment (*p*>0.05). After the treatment, the levels of serum VEGF increased and the levels of ET-1 and MSH decreased in the two groups, but the changes were more pronounced in the study than the control group (*p*<0.05; Table-III).

**Table-III:** Comparison of serum biochemical indexes between the two groups (X̄±*S*).

Status	Group	*n*	VEGF (ng/ml)	ET-1 (pg/ml)	MSH (pg/ml)
Before treatment	Study group	42	87.33±11.53	86.04±15.71	259.71±35.80
	Control group	38	89.31±12.48	88.07±16.01	266.71±41.82
	t		0.738	0.572	0.806
	p		0.463	0.569	0.423
After treatment	Study group	42	110.23±12.53^a^	70.23±12.48^a^	198.57±28.81^a^
	Control group	38	101.44±13.61^a^	78.73±12.21^a^	234.52±37.50^a^
	t		3.007	3.072	4.771
	p		0.004	0.003	<0.001

*Note:*

a indicates a comparison with the group before treatment, p < 0.05.

The incidences of adverse reactions in the study group (7.14%) and the control group (5.26%) were similar (*p*>0.05; Table-IV). Three months after the treatment, the disease recurrence rates were similar in both groups (*p*>0.05). Six months after the treatment, the disease recurrence rate of the study group (4.76%) was lower than that of the control group (21.05%; *p*<0.05; Table-V).

**Table-IV:** Comparison of adverse reaction rates between the two groups [n (%)].

Group	*n*	Depigmentation	Flushed complexion	Scarring	Total incidence
Study group	42	0 (0.00)	2 (4.76)	1 (2.38)	3 (7.14)
Control group	38	1 (2.63)	1 (2.63)	0 (0.00)	2 (5.26)
χ^2^					0.120
p					0.729

**Table-V:** Comparison of disease recurrence rates between the two groups [n (%)].

Group	*n*	Three months after treatment	Six months after treatment
Study group	42	1 (2.38)	2 (4.76)
Control group	38	3 (7.89)	8 (21.05)
Study group		1.277	4.841
p		0.258	0.028

## DISCUSSION

This study evaluated PRP combined with TXA in the treatment of melasma. After six months of treatment, the effect of the combined treatment was significantly higher than that of TXA alone in patients with melasma (*p*<0.05). TXA is a commonly used anticoagulant agent in the clinical practice. Its chemical structure is similar to that of tyrosine, it can compete with plasminogen activator, preventing plasminogen molecules from being transformed into plasmin and thereby inhibiting its proteolytic activity.

TXA can thus inhibit the combination of tyrosinase and tyrosine, and reduce the production of melanin.^11^ At present, a large number of studies have confirmed the safety and reliability of TXA in the treatment of melasma.^3^,^12^ Ebrahim HM et al^13^ studied the treatment of melasma by intradermal injection of TXA and TXA microneedle. They showed that both methods are safe and effective, but microinjection of TXA is more satisfactory to patients, thus becoming one of the research directions of the follow-up team. However, there are still many studies that show the limitation of the treatment with tranexamic acid alone.

Minni K et al^14^ showed that TXA has a definite effect on melasma and is a promising treatment method. However, after the 24^th^ week of treatment with oral TXA alone, researchers observed a melasma recurrence rate of 18.03%. In our study, the adverse reaction rate of patients taking TXA tablets alone for three months was 21.05%.

PRP has been found to contain a large amount of fibrin, leukocytes, and platelets. After platelet activation, these cells can produce a variety of growth factors, including epidermal growth factor and VEGF. These growth factors are interrelated and can activate signal pathways that promote cell proliferation and matrix formation.^15^ Sirithanabadeekul P et al^16^ confirmed the beneficial effects of PRP in the treatment of melasma with high patient satisfaction levels. The study of Tuknayat A et al^17^ showed that PRP contains approximately 30 growth factors.

Transforming growth factor β (TGF-β) is one of the main growth factors for the treatment of melasma. PRP can be used as an adjuvant treatment or as an independent treatment against melasma. We retrospectively analyzed the clinical data of patients with melasma treated with PRP and TXA or with TXA alone to compare the therapeutic effects against melasma between the two groups. Our results show that the total efficacy in the study group was higher than that in the control group, and that the disease recurrence rate six months after the treatment was lower in the study group (*p*<0.05) with similar adverse reaction incidences.

Gamea MM *et al*^18^ conducted an RCT including 40 patients divided into two groups, and showed that the treatment effect and patient satisfaction of PRP combined with TXA group were significantly higher than those of TXA alone group. The growth factors, fibrin and leukocytes in the PRP can be injected using water light injection into the skin tissue to regulate and repair the whole layer structure of the skin, improving the skin barrier function and also rebuilding the skin microcirculation, promoting skin pigment metabolism and fading away the hyperpigmented spots of melasma.^19^ Moreover, PRP has bacteriostatic, anti-inflammatory and repair functions that contribute to the metabolism of the abnormal hyperpigmentation observed in melasma.^20^

The pathogenesis of melasma is associated with abnormal microcirculation *in vivo*. VEGF, a vascular growth factor, can promote angiogenesis and endothelial proliferation, and it can improve vascular permeability. In addition, VEGF stimulates production of other vascular growth factors.^21^ Imokawa et al^22^ showed that ET-1 is a mitotic agent for melanocytes that can accelerate their proliferation, improving their tyrosinase activity and up regulating the production of melanin. Studies have also shown that MSH is mainly produced in the pituitary interstitium. It can activate tyrosinase, accelerate the production of melanin and tyrosinase, reduce the level of internal sulfhydryl groups, increase the content of copper ion and promote pigment synthesis.^23^ Our results show that after the treatment, the levels of VEGF were higher and the levels of ET-1 and MSH were lower in the study group compared to the control group (*p*<0.05), confirming the clinical value of PRP combined with TXA in the treatment of melasma. In all, treatment with PRP combined with TXA seems to effectively upregulate the level of VEGF and downregulate the serum expression of ET-1 and MSH in patients with melasma. This may explain the improved results seen with the combined therapy as compared to the results after the TXA alone.

### Limitations of the study:

This was a single center study with a small sample size, a short follow-up time of only six months and few observation indicators. The factors affecting melasma may include inflammation, reactive oxygen species, ultraviolet radiation, genetic factors, and hormones. With the further understanding of the pathogenesis of melasma, some scholars have proposed the combined treatment scheme of hydroquinone, anti-estrogen and vascular endothelial growth factor inhibitors,^3^ and laser assisted tranexamic acid treatment,^24^ which are also the future research direction of our team.

## CONCLUSION

PRP combined with TXA in the treatment of melasma can improve the treatment outcome safely regulating the levels of VEGF, ET-1 and MSH and reducing the recurrence rate.

### Authors’ contributions:

**CZ** conceived and designed the study.

**TW and NS** collected the data and performed the analysis.

**CZ** was involved in the writing of the manuscript and is responsible for the integrity of the study.

All authors have read and approved the final manuscript.
